# Editorial: Polymeric biomaterials for regenerative medicine

**DOI:** 10.3389/fbioe.2023.1297865

**Published:** 2023-09-26

**Authors:** Zhenlin Yang, Dingpei Long

**Affiliations:** ^1^ State Key Laboratory of Resource Insects, Institute of Sericulture and Systems Biology, Southwest University, Chongqing, China; ^2^ Institute for Biomedical Sciences, Center for Inflammation, Immunity and Infection, Digestive Disease Research Group, Georgia State University, Atlanta, GA, United States

**Keywords:** polymer biomaterial, tissue regeneration, scaffolds, coating, membranes, hydrogel, drug delivery

The progress in regenerative medicine and tissue engineering has introduced new challenges for biomaterials. In addition to requiring biosafety and high biocompatibility, implanted biomaterials are now expected to deliver drugs with precision and efficiency, along with controlled release capabilities. Consequently, the development of polymeric biomaterials with specific biological functions for regenerative medicine has garnered significant attention. This objective of this Research Topic is to present the latest advancements in the design, manufacturing, and *ex vivo* assessment of polymer biomaterials for tissue regeneration. This Research Topic includes five papers focusing on various polymer biomaterials such as hydrogels, scaffolds, composite nanoparticles, and microneedle patches. These papers encompass one review article and four original research articles, and a brief description of each paper is provided below.

Secondary spinal cord injury (SSCI) is associated with vascular complications, inflammatory responses, and the formation of neuroglial scars. In recent years, the utilization of hydrogels as scaffolds and drug delivery systems for the treatment of SSCIs has gained significant traction and is progressively making its way into clinical applications (Madigan et al., 2014; Silva et al., 2021). The review authored by Peng et al. comprehensively outlines the pathophysiology of SSCI and delves into the remarkable potential of hydrogels as scaffolds and delivery systems in SSCI treatment. The review highlights how hydrogels can serve as scaffolds to facilitate neuronal cell and axon growth, emphasizing specific attributes such as adhesion, oriented alignment, structural integrity, electrical conductivity, and injectability that greatly enhance hydrogels’ efficacy as scaffolds and drug delivery platforms. Furthermore, the integration of synthetic polymers enhances the mechanical properties of natural hydrogels, and advancements in fabrication methods, such as three-dimensional printing and electrostatic spinning, have further refined their utility. Moreover, chemically and physically modified hydrogels offer additional functionalities, expanding their applicability in SSCI therapy and presenting promising prospects for clinical use.

The ideal scaffold for pulp regeneration has been identified as the decellularized extracellular matrix (ECM). However, a significant challenge in the field of pulp regulation science has been the absence of composite ECM scaffolds capable of systematically regulating coordinated tissue regeneration and enhancing revascularization in the functional pulp-dentin complex (Raddall et al., 2019). Additionally, the limited availability of natural pulp tissue restricts its clinical application. To address these challenges and compare the pulp regeneration properties of two scaffolds, Shi et al. successfully decellularized rat submandibular gland and human dental pulp. Their findings demonstrated that the decellularized submandibular gland extracellular matrix (DSMG) played a role in promoting dentinogenesis and angiogenesis, while also supporting the proliferation and adhesion of pulp stem cells *in vitro*. In conclusion, their study introduced a novel technique for developing an ECM-based scaffold material, which is predicted to have a significant impact on pulp regeneration therapy.

Due to the existing limitations of biomaterials in bone regeneration, such as their osteogenic and antimicrobial properties (Qian et al., 2019), research in bone tissue engineering has become crucial for investigating effective biomaterials for drug delivery in osteogenic repair. In a study conducted by Chen et al., they utilized magnetic stirring to create metformin/human serum albumin (HSA)/chitosan nanoparticles (MHCNPs) by mixing HSA, metformin hydrochloride, and chitosan. The aim was to evaluate the *in vitro* therapeutic benefits of this nanopreparation on bone injuries. To assess the osteoinductive capabilities of MHCNPs, the study stimulated the differentiation of mouse bone marrow mesenchymal stem cells (BMSCs) into neuronal cells *in vitro*. This evaluation was carried out by measuring alkaline phosphatase activity and employing real-time fluorescence quantitative PCR to detect the expression of osteocalcin and osteoprotectin genes associated with osteogenesis in BMSCs. The findings revealed that osteoinductive properties of MHCNPs exhibited favorable attributes such as stability, biocompatibility, and biodegradability. Moreover, they demonstrated the sustained release of dimethylene bis-arc, promoting osteogenesis over time. As synthetic biopolymers derived from these nanoparticles, MHCNPs hold great promise as an excellent drug delivery system and are expected to play a crucial role in the repair of bone tissue.

As reported in Jiang et al. (2022), the M2 subtype of macrophages is activated and polarized by the left-handed chiral matrix. However, prior to this study, there had been no research examining how the chiral microenvironment influences the proteome of macrophages *in vivo* during bone defect repair. In a separate investigation, Zeng et al. established a rat cranial bone defect model and conducted proteomic analysis of proteins extracted from the bone defect areas that were implanted with both levo- and dextro- (right-handed) scaffolding matrices during the early healing phase. Protein sequencing was performed at both the 3-day and 7-day time points. The results showed that levo-chirality upregulated cell adhesion-associated proteins on day 3 and continued to upregulate GTPase-associated proteins on day 7. Furthermore, interaction analysis and *in vitro* validation results revealed the first evidence that chirality could lead to differential activation of Rho GTPases and downstream Akt1. This ultimately resulted in heterogeneity in macrophage polarization, shedding light on the variations in bone repair outcomes. This research contributes to a better understanding of the role of chirality in macrophage behavior and its implications for bone regeneration.

Drug delivery methods for addressing oral submucosal fibrotic degeneration encompass both local administration and submucosal injection. However, these approaches have limitations, including restricted local administration and discomfort associated with injections. Therefore, there is a pressing need to develop an alternative local drug delivery system that is both efficient and painless (Cox and Zoellner, 2009; Alqahtani et al., 2021). In response to this challenge, Cheng et al. introduced a novel two-layer mucosal-adherent microneedle patch for transmucosal drug delivery. This patch consists of a mucosal-adherent top layer containing filipin and tannic acid, along with a filipin microneedle bottom layer. In comparison to commercially available oral patches, the bilayer patch exhibited significantly enhanced wet adhesion strength of 37.74 kPa. Furthermore, *ex vivo* mucosal tissue permeation confirmed the patch’s ability to reach the lamina propria, ensuring effective drug delivery to the lesion site of oral submucosal fibrotic degeneration. These findings hold great promise for the use of drug-loaded mucosal adhesion microneedle patches in the treatment of oral submucous fibrous lesions.

Overall, this topical research theme encompasses five articles exploring the roles played by polymeric biomaterials with diverse properties in tissue regeneration. These articles showcase the valuable contributions of experts from various fields to the advancement of biomaterials and regenerative medicine therapeutics. Collectively, this compilation of articles offers readers a comprehensive overview of the field of polymeric biomaterials and is sure to spark their interest in this exciting area of polymer biomaterial research.
